# Non-communicable respiratory disease and air pollution exposure in Malawi: a prospective cohort study

**DOI:** 10.1136/thoraxjnl-2019-213941

**Published:** 2020-02-20

**Authors:** Sarah Rylance, Chris Jewell, Andrew Naunje, Frank Mbalume, John D Chetwood, Rebecca Nightingale, Lindsay Zurba, Graham Flitz, Stephen B Gordon, Maia Lesosky, John R Balmes, Kevin Mortimer

**Affiliations:** 1 Department of Clinical Sciences, Liverpool School of Tropical Medicine, Liverpool, UK; 2 Lung Health Group, Malawi Liverpool Wellcome Trust Clinical Research Programme, Blantyre, Malawi; 3 CHICAS, University of Lancaster, Lancaster, UK; 4 John Hunter Hospital, New Lambton Heights, New South Wales, Australia; 5 Education for Health Africa, Vereeniging, South Africa; 6 School of Public Health, University of California, Berkeley, California, USA; 7 Division of Epidemiology and Biostatistics, School of Public Health and Family Medicine, University of Cape Town, Rondebosch, Cape Town, South Africa; 8 Environmental Health Sciences Division, University of California San Francisco, San Francisco, California, USA; 9 Aintree University Hospitals NHS Foundation Trust, Liverpool, UK

**Keywords:** clinical Epidemiology, lung Physiology

## Abstract

**Rationale:**

There are no population-based studies from sub-Saharan Africa describing longitudinal lung function in adults.

**Objectives:**

To explore the lung function trajectories and their determinants, including the effects of air pollution exposures and the cleaner-burning biomass-fuelled cookstove intervention of the Cooking and Pneumonia Study (CAPS), in adults living in rural Malawi.

**Methods:**

We assessed respiratory symptoms and exposures, spirometry and measured 48-hour personal exposure to fine particulate matter (PM_2.5_) and carbon monoxide (CO), on three occasions over 3 years. Longitudinal data were analysed using mixed-effects modelling by maximum likelihood estimation.

**Measurements and main results:**

We recruited 1481 adults, mean (SD) age 43.8 (17.8) years, including 523 participants from CAPS households (271 intervention; 252 controls), and collected multiple spirometry and air pollution measurements for 654 (44%) and 929 (63%), respectively. Compared with Global Lung Function Initiative African-American reference ranges, mean (SD) FEV_1_ (forced expiratory volume in 1 s) and FVC (forced vital capacity) z-scores were −0.38 (1.14) and −0.19 (1.09). FEV_1_ and FVC were determined by age, sex, height, previous TB and body mass index, with FEV_1_ declining by 30.9 mL/year (95% CI: 21.6 to 40.1) and FVC by 38.3 mL/year (95% CI: 28.5 to 48.1). There was decreased exposure to PM_2.5_ in those with access to a cookstove but no effect on lung function.

**Conclusions:**

We did not observe accelerated lung function decline in this cohort of Malawian adults, compared with that reported in healthy, non-smoking populations from high-income countries; this suggests that the lung function deficits we measured in adulthood may have origins in early life.

**Key messages**

**What is the key question?**

- Are the low lung volumes previously reported in adults from Malawi a result of impaired lung development in early life or accelerated lung function decline in adulthood or both, and does biomass smoke exposure influence the rate of decline in the same way as tobacco smoke exposure?

**What is the bottom line?**

- In an adult population with high biomass smoke exposure, we found rates of lung function decline comparable with healthy non-smokers in high-income countries and lung function z-scores consistent with those reported in children from the same rural Malawian community.

**Why read on?**

- We report the first longitudinal lung function data from a population-representative cohort in sub-Saharan Africa: the results suggest that exposure to biomass fuel smoke may be less harmful than exposure to tobacco smoke or traffic-related air pollution, as reported in high-income settings.

## Introduction

Non-communicable respiratory diseases including chronic obstructive pulmonary disease (COPD) and asthma are a growing global concern, particularly in low-income and middle-income countries.^1–3^ Air pollution, including exposure to tobacco smoke, outdoor and household air pollutants, and occupational exposure to dust and fumes, is considered a major risk factor for non-communicable respiratory disease development and exacerbations.^1 4^ However, conflicting findings from recent studies have cast uncertainty over the specific role of household air pollution in COPD development.^5 6^ Approximately 3 billion people worldwide rely on highly polluting biomass fuels for cooking, heating and lighting.^7^ It is therefore a global public health priority to better understand the impact of household air pollution on non-communicable respiratory disease morbidity and mortality.

The lung function trajectories of adults from sub-Saharan Africa (sSA) are largely undescribed; limited published data relate to cohorts from South Africa with HIV-infection and occupational silica dust exposure.^8 9^ There are no data from population-representative cohorts in sSA; it is not known whether adults exposed to biomass-related air pollution would experience accelerated age-related decline in lung function and therefore an increased risk of developing obstructive airways diseases as occurs in those exposed to tobacco smoke.^10 11^

The cross-sectional BOLD (Burden of Obstructive Lung Disease) study, conducted in urban Blantyre, Malawi found unexpectedly high rates of decreased forced vital capacity (FVC) and high levels of self-reported exposure to biomass smoke.^12^ The finding of a high burden of low FVC was concerning given the association between this and increased mortality.^13^ To further explore this phenomenon, we did a second study in rural Chikhwawa, Malawi (entitled BOLD-Chikhwawa) with the same protocol as the Blantyre BOLD study, but with the addition of measurement of personal exposure to air pollutants: carbon monoxide (CO) and fine particulate matter <2.5 µm (PM_2.5_).^14^ We found comparably high rates of spirometric abnormalities, with decreased FVC seen in 35% of participants, but no association between spirometric outcomes and exposure to CO or PM_2.5_ despite high levels of air pollution. Participants were from village communities which also participated in the Cooking and Pneumonia Study (CAPS), a cluster randomised trial of a cleaner-burning biomass-fuelled cookstove.^15^ Secondary analysis of adults from a subset of CAPS households found no difference in respiratory symptoms, lung function or personal air pollution exposures between intervention and control groups, but these analyses were done using cross-sectional data that were collected only a short time after introduction of the intervention—it is not known whether the rate of decline in lung function over time would be different between the trial arms.^14^

In this paper, we report the findings of lung function and personal air pollutant exposure monitoring during 3 years of follow-up for the BOLD-Chikhwawa cohort, to explore the determinants of lung function trajectories, including the effect of the CAPS cookstove intervention, in adults living in rural Malawi.

## Methods

### Setting

Chikhwawa is a rural district, approximately 50 km south of Blantyre, on the Shire River valley. During the study period, this district experienced severe flooding and crop failures. CAPS recruited children aged <4.5 years in Chikhwawa between December 2013 and August 2015; intervention households received two cleaner-burning biomass-fuelled cookstoves, a solar panel to charge the stove-fan battery and user training at the time of randomisation. Those in the control arm continued using traditional cooking methods, mostly open fires, but received cookstoves at the end of the CAPS follow-up in May 2016.

**Figure 1:**
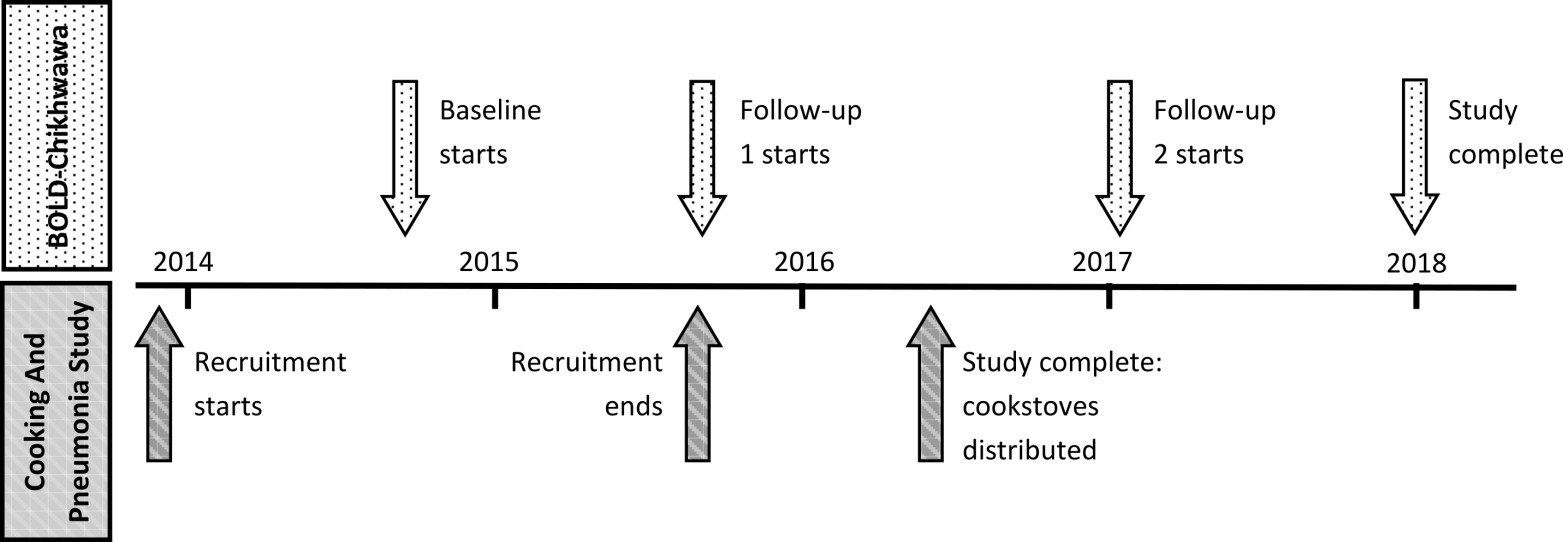
Timeline showing CAPS and BOLD-Chikhwawa activities. BOLD, Burden of Obstructive Lung Disease; CAPS, Cooking and Pneumonia Study.

BOLD-Chikhwawa was a separate study, recruiting adults from the same village communities as CAPS: not all BOLD-Chikhwawa participants were enrolled in CAPS. Figure 1 shows the timeline of CAPS and BOLD-Chikhwawa activities.

### Participants

Age-stratified and gender-stratified population-representative sample of adults from 50 villages in Chikhwawa was taken as previously described.^14^ Written informed consent (or witnessed thumbprint) was obtained, with the information provided in the local language, Chichewa.

### Procedures

Fieldworkers conducted follow-up visits in the community, approximately 1 and 2 years after enrolment, according to BOLD study standardised operating procedures, to collect questionnaires, spirometry and personal air pollution exposure data.^16^ Fieldworkers administered an abbreviated version of the BOLD study questionnaire in Chichewa, and measured height and weight.

BOLD Centre-certified fieldworkers conducted spirometry according to European Respiratory Society(ERS)/American Thoracic Society (ATS) standards using an EasyOne Spirometer (ndd Medical Technologies; Zurich, Switzerland), before and after administration of 200 μg of Salbutamol via Volumatic spacer.^17^ Spirometry overreading was performed by an independent technician, according to the BOLD criteria for acceptability and repeatability.

After completing the questionnaire and spirometry assessment, participants were given an Indoor Air Pollution (IAP) 5000 Series Monitor (Aprovecho Research Centre, Oregon, USA) which they were instructed to wear in a small backpack during the day and to keep beside their sleeping mat at night, to estimate personal exposure to PM_2.5_ and CO over a 48-hour period. These monitors continuously sample air from the breathing zone, and PM_2.5_ and CO are measured using a light-scattering photometer and electrochemical cell CO sensor, respectively. Fieldworkers encouraged compliance with personal exposure monitoring during frequent community visits. IAP traces with outlying PM_2.5_ or CO values (extremely high or low) were visually inspected for expected daily variation in exposure: traces without variability, suggesting that backpacks had not been worn, were excluded from the analysis.

### Variables

Clinical outcomes were assessed by the questions detailed in table 1. Raw forced expiratory volume in 1 s (FEV_1_) and FVC values were used as continuous variables in the longitudinal analysis. Lung function parameters were compared with age, sex and height-standardised global lung initiative (GLI) reference ranges for African-Americans and NHANES III reference ranges for Caucasians and African-Americans.^18 19^ Restriction was defined as FVC below the lower limit of normal (LLN), and obstruction as FEV_1_/FVC ratio below the LLN; values below the fifth centile in a healthy, non-smoking reference population.

**Table 1:** Respiratory symptoms and exposures reported by participants at baseline, first and second follow-up.

	% (95% CI)		
	Baseline (n=1481)	Follow-up 1 (n=1090)	Follow-up 2 (n=989)
Respiratory symptoms			
Cough: Do you usually cough when you do not have a cold?	11.1 (9.6 to 12.9)	10.1 (8.4 to 12.0)	25.3 (22.6 to 28.1)
Sputum: Do you usually bring up phlegm from your chest when you do not have a cold?	2.6 (1.8 to 3.5)	4.9 (3.7 to 6.3)	11.1 (9.2 to 13.2)
Wheeze: Have you had wheezing/whistling in your chest in the last 12 months, in the absence of a cold?	1.6 (1.0 to 2.3)	1.7 (1.0 to 2.6)	3.0 (2.1 to 4.3)
MRC dyspnoea II: Are you troubled by shortness of breath when hurrying on the level or walking up a slight hill?	1.6 (1.0 to 2.3)	6.6 (5.2 to 8.2)	11.8 (9.9 to 14.0)
Functional limitation: Have breathing problems interfered with your usual daily activities?	2.9 (2.1 to 3.9)	5.7 (4.4 to 7.2)	7.1 (5.6 to 8.9)
Any respiratory symptom (any of the above five symptoms)	13.6 (11.9 to 15.4)	19.6 (17.3 to 22.1)	36.2 (33.3 to 39.4)
Self-reported exposures			
Current smoker	13.9 (12.2 to 15.8)	11.6 (9.7 to 13.6)	12.9 (10.9 to 15.2)
Previous TB	3.2 (2.3 to 4.2)	3.0 (2.1 to 4.2)	2.6 (1.7 to 3.8)

TB, tuberculosis.

Exposures included estimated personal exposure to PM_2.5_ and CO, and questionnaire assessment of smoking status and previous tuberculosis. At baseline, first and second follow-up, participants were classed as having access to a cookstove if their household had been given a cleaner-burning biomass-fuelled cookstove by the CAPS study team prior to data collection.

Baseline PM_2.5_ and CO levels were zeroed at the 0.1th percentile of values obtained during each monitoring period. Observations were included if >24 hours were recorded, with recording truncated into 24-hour periods to reflect daily variation in personal exposure patterns, and only full 24-hour periods analysed. Log mean 24-hour PM_2.5_ and CO estimates were used for mixed-effects modelling.

Potential effect modifiers: body mass index (BMI) and/or height and weight, age, years of education and sex, were evaluated as fixed covariates in the FEV_1_ and FVC linear mixed-effects models.

### Study size

A total of 3000 adults were initially invited to enrol in the baseline BOLD-Chikhwawa cohort. Participants were followed up if they had completed a baseline questionnaire (1481 participants) and were included in the longitudinal lung function analysis if they had at least two valid spirometry assessments during the study period.

### Statistical methods

Descriptive analysis was performed, with the Student *t* test and Pearson’s χ^2^ to compare continuous and categorical data.

Participants with incomplete data (lost-to-follow-up or failing to complete spirometry) were compared with those with complete data using χ^2^ and Student *t* tests. Positive associations (p<0.2) on bivariate analysis were explored in multivariable logistic regression.

Two separate mixed-effects models were developed for the analysis of repeated exposure and lung function outcomes. In the log-linear exposure models, repeated estimates (mean 24 hours CO and PM_2.5_) from individuals were accounted for using an individual-level random effect, with an additional random-effect accounting for clustering of 24-hour measurements within 48-hour monitoring periods. Fixed-effect covariates were selected sequentially to determine the optimum model fit by likelihood ratio testing under maximum likelihood estimation (MLE), with the calculation of parameter estimates, standard errors and p values (see the online supplementary tables S2 and S3). Harmonic terms were included in the exposure models to account for any possible effect of seasonality on the outcome measures. This was implemented by including sinusoidal functions (sine and cosine terms) of time with a period of 1 year.

10.1136/thoraxjnl-2019-213941.supp1 Supplementary data

Longitudinal lung function (FEV_1_ and FVC) linear models included the fitted CO and PM_2.5_ values from the exposure model as fixed covariates; an average value was calculated where participants had multiple periods of exposure monitoring. Fixed-effect covariates were sequentially assessed by likelihood ratio testing under MLE (see the online supplementary tables S4 and S5), with interaction terms to explore the change in lung function over time. The final regression equations used in the exposure and lung function analysis are included in the online supplementary text S1.

Analyses were conducted using R V.3.4.1 statistical software.

## Results

**Figure 2:**
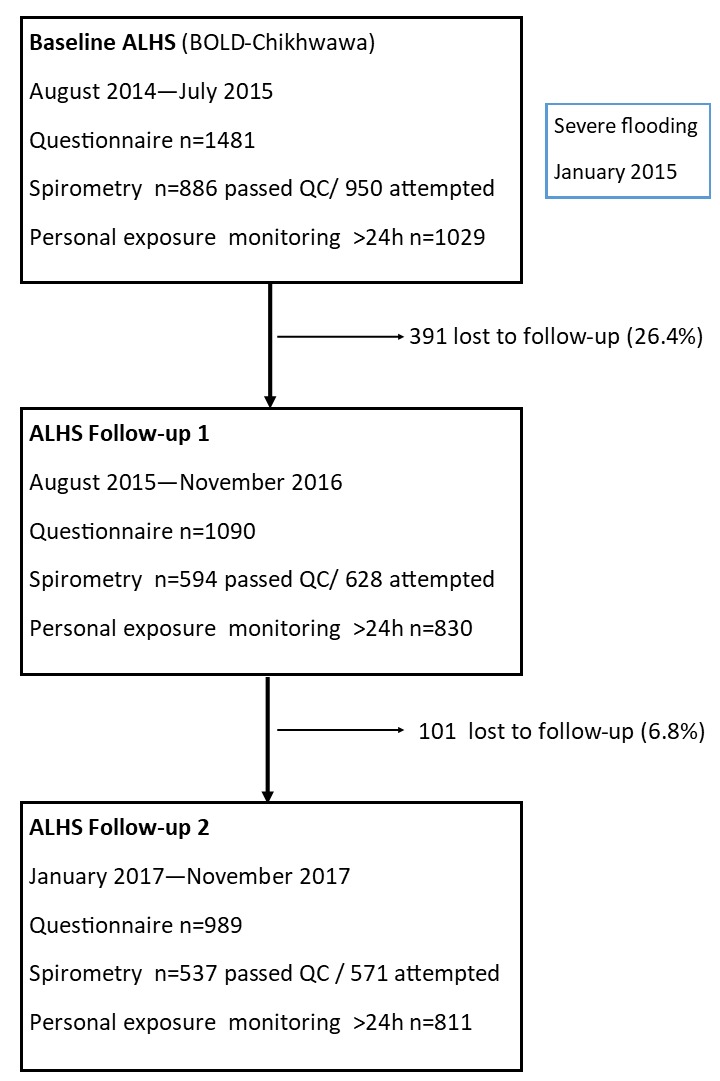
Participant flow diagram.

Between August 2014 and July 2015, 1481 adults were enrolled in the study at baseline and followed up on two subsequent occasions.^14^ Three-quarters (75%, n=1090) were reassessed during the first follow-up period (August 2015–November 2016) and two-thirds (67%, n=989) during the second follow-up period (January 2017–November 2017) with data collected as shown in figure 2. Demographic data for participants with or without a questionnaire, spirometry or exposure monitoring are shown in the online supplementary table S1. Participants completing the second follow-up visit were more likely to be women (OR (95% CI): 1.88 (1.50 to 2.37), and to have spent fewer years in education (OR (95% CI): 0.96 (0.93 to 0.99)).

Spirometry was attempted by 950/1481 (64%), 628/1090 (58%) and 571/989 (58%), and personal air pollution exposure monitoring completed for 1029/1481 (69%), 830/1090 (76%) and 811/989 (82%) at baseline, first and second follow-ups, respectively (figure 2). Multiple spirometry measurements were available for 654/1481 (44%) of participants, whereas 413 (28%) had only one spirometry measurement and 413 (28%) had none. Personal air pollution exposure was estimated on more than one occasion for 929/1481 (63%) of participants, whereas 401 (27%) had only one episode of monitoring and 151 (10%) had none.

At baseline, the cohort included 424 participants from CAPS households: this rose to 523 participants (271 from intervention and 252 from control households) as CAPS continued to recruit until August 2015.

The baseline demographics of the cohort have been previously reported.^14^ In brief, at baseline the mean (SD) age of participants was 43.8 (17.8) years, 57% were women and all households (99.8%) used biomass fuels for cooking. One third had never attended school and half had not been educated beyond the primary school level.

The frequency of reported respiratory symptoms increased greatly over the course of the study (table 1): overall 13.6% (95% CI: 11.9 to 15.4) of participants reported respiratory symptoms at baseline compared with 36.2% (95% CI: 33.3 to 39.4) at the final follow-up. Self-reported rates of smoking and TB did not change over time; current smoking was reported by 13.9% and 12.9%, and previous TB infection reported by 3.2% and 2.6%, at the baseline and final follow-up, respectively.

### Personal exposure monitoring

A total of 1768 personal exposure monitoring episodes lasted >48 hours, and a further 902 lasted between 24 and 48 hours. Within episodes of >48 hours, there was fair correlation between the first and second 24-hour periods, for both PM_2.5_ (adjusted *R* ^2^=0.68) and CO (adjusted *R* ^2^=0.59). Correlation between exposures to the two air pollutant measures (mean PM_2.5_ and CO), analysed for a total of 4438 24 hours monitoring periods was poor (adjusted *R* ^2^=0.027).

Overall, the 24 hours median personal PM_2.5_ and CO exposures were 77.0 µg/m^3^ (IQR, 42.8–153.1) and 1.27 ppm (IQR, 0.79–2.05), respectively. Personal PM_2.5_ (median (IQR)) was 71.7 µg/m^3^ (42.8–128.0), 84.6 µg/m^3^ (45.9–175.7) and 75.9 µg/m^3^ (40.1–176.4) at baseline, first and second follow-up, respectively. Personal CO exposure (median (IQR)) was 1.26 (0.79–2.07) ppm, 1.33 (0.81–2.22) ppm and 1.22 (0.75–1.90) ppm, at baseline, first and second follow-up, respectively.

In total, 4377 24-hour monitoring periods with complete covariate data from 1304 individuals were included in mixed-effects exposure models, with CO and PM_2.5_ as the response variables. In the final CO model, we found strong evidence that female sex, current smoking status and seasonality were associated with the CO level (tables 2 and online supplementary table S2). In the final PM_2.5_ model, female sex was associated with increased PM_2.5_ and access to a cookstove with decreased PM_2.5_ (risk ratio 0.85; 95% CI: 0.75 to 0.97) (tables 2 and online supplementary table S3).

**Table 2:** Estimated risk ratios and 95% CIs for fixed-effects covariates included in final air pollutant exposure log-linear mixed-effect models

	PM_2.5_ (µg/m^3^)	CO (ppm)
Sex	1.27 (1.13 to 1.42)	1.60 (1.51 to 1.72)
Current smoking	–	1.22 (1.12 to 1.34)
Seasonality: cosine function	–	0.85 (0.81 to 0.89)
Seasonality: sine function	–	0.99 (0.96 to 1.03)
Access to cookstove	0.85 (0.75 to 0.97)	–

CO, carbon monoxide; PM_2.5_, fine particulate matter.

### Spirometry

Of those attempting spirometry, ERS/ATS standards were achieved by 886/950 (93.3%), 594/628 (94.6%) and 537/571 (94.0%) at baseline, first and second follow-up visits, respectively (figure 2). On bivariate analysis, factors associated with failing to complete spirometry were: older age, lower BMI, female sex, current smoking, cough or any respiratory symptoms. In logistic multivariable analysis, participants who were women (OR (95% CI), 0.52 (0.39–0.71)), older (OR (95% CI), 0.97 (0.96–0.98)) or with a lower BMI (OR (95% CI), 1.09 (1.04–1.14)) were significantly less likely to complete spirometry. Participants with longitudinal spirometry data had reduced lung function at baseline, compared with those who performed spirometry on only one occasion: mean (SD) FEV z-score −0.48 (1.03) vs -0.22 (1.28), mean (SD) FVC z-score −0.33 (1.01) vs 0.03 (1.19), respectively.

Best post-bronchodilator traces were analysed for 1068 participants who completed at least one spirometry session to ERS/ATS standards. Overall, mean (SD) FEV_1_ and FVC were 2.55 (0.64) L and 3.16 (0.73) L, respectively, with a mean (SD) FEV_1_/FVC ratio of 0.80 (0.09) (table 3). When compared with GLI African-American reference ranges, mean (SD) FEV_1_, FVC and FEV_1_/FVC ratio z-scores were −0.38 (1.14), –0.19 (1.09) and −0.37 (1.04), respectively, with spirometric obstruction seen in 11.2% (95% CI: 9.4% to 13.2%) and low FVC in 8.1% (95% CI: 6.5% to 9.9%). Rates of obstruction were similar when NHANES Caucasian reference ranges were used (11.5% (95% CI: 9.6% to 13.5%)), but considerably more—approximately 50%—of participants were classified as having a low FVC (49.7% (95% CI: 46.7% to 52.8%).

**Table 3:** Best post-bronchodilator spirometry values* and classification by GLI and NHANES reference ranges for 1068 participants

Spirometry value		
Raw	Mean (SD) FEV_1_, L	2.55 (0.64)
	Mean (SD) FVC, L	3.16 (0.73)
	Mean (SD) FEV_1_/FVC ratio	0.80 (0.09)
Z-scores†	Mean (SD) FEV_1_ z-score	−0.38 (1.14)
	Mean (SD) FVC z-score	−0.19 (1.09)
	Mean (SD) FEV_1_/FVC ratio z-score	−0.37 (1.04)

Classification		% of Population (95% CI)
Obstruction	FEV_1_/FVC<LLN GLI African-American	11.2 (9.4 to 13.2)
	FEV_1_/FVC<LLN NHANES African-American	11.5 (9.6 to 13.5)
	FEV_1_/FVC<LLN NHANES Caucasian	9.8 (8.1 to 11.7)
Restriction	FVC <LLN GLI African-American	8.1 (6.5 to 9.9)
	FVC <LLN NHANES African-American	7.7 (6.2 to 9.5)
	FVC <LLN NHANES Caucasian	49.7 (46.7 to 52.8)

†Z-scores calculated using GLI African-American reference ranges.

*For participants with spirometry measured at more than one time point, the best FEV_1_ and FVC values were analysed.

FEV_1_, forced expiratory volume in 1 s; FVC, forced vital capacity; GLI, global lung initiative; LLN, lower limit of normal.

Overall, the annual rate of lung function decline was 30.9ml (95% CI: 21.6 to 40.1) for FEV_1_ and 38.3ml (95% CI: 28.5 to 48.1) for FVC. Age, sex, height, previous TB infection and BMI were included in the final mixed-effects models as significant fixed-effect covariates for FEV_1_ and FVC (all p<0.001, online supplementary table S4 and S5), although they did not affect the rate of lung function decline. Current smoking, access to a cookstove, PM2.5 and CO exposure levels did not significantly improve either model. Decreased FEV_1_ and FVC were associated with increasing age, female sex, previous TB infection and decreased height and BMI (table 4).

**Table 4:** Parameter estimates for multiple fixed-effects covariates included in final FEV_1_ and FVC linear mixed-effect models*

	FEV_1_ (mL)		FVC (mL)	
	Estimate	95% CI	Estimate	95% CI
Time (years)	−30.9	−40.1 to 21.6	−38.3	−48.1 to −28.5
Age (years)	−18.7	−20.4 to −16.9	−11.0	−13.0 to −9.1
Sex (female)	−500.1	−566.6 to −433.6	−678.0	−751.4 to −604.7
Height (cm)	23.6	19.9 to 27.3	32.8	28.7 to 36.9
Previous TB (yes)	−404.9	−539.7 to −230.2	−334.2	−526.6 to −141.8
BMI	21.9	13.8 to 30.0	21.3	12.4 to 30.2

*Models include FEV_1_ and FVC data from 950 individuals, including 654 with two or more lung function measurements

BMI, body mass index; FEV_1_, forced expiratory volume in 1 second; FVC, forced vital capacity; TB, tuberculosis.

## Discussion

This is the first prospective cohort study to report longitudinal lung function and personal exposure to air pollution in an sSA population. The main findings were that: FEV_1_ and FVC were determined by age, sex, height, previous TB and BMI, whereas there was no evidence of accelerated lung function decline (30.9 mL FEV_1_ and 38.3 mL FVC annual decrease) as might have been expected in this population compared with the natural age-related decline reported in populations from Europe and the USA.^10^ Mean (SD) FEV_1_ and FVC z-scores (−0.38 (1.14) and −0.19 (1.09)) were comparable with those previously reported for children from this community adding to evidence that spirometric abnormalities in adults have their origins in early life.^20^ Lung function was not associated with exposure to CO, PM_2.5_ or access to a cookstove. Estimated CO and PM_2.5_ correlated poorly and were associated with different covariates. Exposure to PM_2.5_ was increased in women and decreased by a factor of 0.85 (95% CI: 0.75 to 0.97) in those with access to a cookstove. Exposure to CO was increased in women and current smokers and showed a seasonal trend.

We did not find evidence of accelerated lung function decline despite exposure to high levels of PM_2.5_. Previous studies exploring the impact of PM_2.5_ on lung function in high-income settings have focused on PM_2.5_ from ambient air pollution, particularly traffic-related air pollution (TRAP). Faster lung function decline was associated with increasing PM_2.5_ in longitudinal cohorts from the USA and Taiwan, the effects of other pollutants were not reported.^21 22^ A large multicentre metanalysis from the European ESCAPE cohorts did not find an association between air pollution and lung function decline but noted that NO_2_ was negatively associated with lung function.^23^ It is possible that the emissions from incomplete biomass combustion are less harmful to the airways than the many constituents (including nitrogen oxides) of TRAP.

Previous work from Malawi has reported lung function relative to NHANES III Caucasian reference values to facilitate comparison with other BOLD studies.^12 14^ In this analysis, we have additionally compared our data with African-American reference ranges (NHANES and GLI). The prevalence of reduced FVC varies greatly depending on which reference equation is used.^24^ The prognostic significance of markedly different predicted values in different ethnic populations is unclear.^25^ Reduced FVC is seen in restrictive lung disease, however a more detailed assessment of total lung capacity by plethysmography is needed to further characterise the pattern of lung defect seen in African populations.

The use of GLI reference ranges permitted direct comparison with spirometry data from children living in the same community. We recently reported lung function for children aged 6–8 years, living in Chikhwawa; FEV_1_, FVC and FEV_1_/FVC ratio z-scores were −0.48 (0.93),–0.30 (0.96) and −0.38 (0.90), respectively, compared with GLI African-American reference ranges.^20^ The finding of similar z-scores in both the children and adults living in this community suggests that factors which influence lung growth and development act in early childhood before 6 years of age, perhaps even starting in-utero.

We found an increase in self-reported respiratory symptoms over the 3-year follow-up period but no changes in exposures (self-reported TB or smoking status, or measured PM_2.5_ or CO). We speculate this is due to changes in reporting behaviour rather than a true change in symptom prevalence. During the CAPS period, the local community were exposed to messages about the health impact of air pollution and may have become sensitised to the issues of clean air and respiratory health. Participants became familiar with the same questions asked on repeated occasions: this may have led to a positive reporting bias. Alternatively, responses at baseline may have underreported symptom prevalence: a community survey in two rural districts in Central Malawi reported chronic respiratory symptoms in 22.5% of the population.^26^

Previous cookstove intervention trials have explored lung function in adult women only.^27 28^ The RESPIRE randomised controlled trial in Guatemala reported a reduction in 48-hour personal CO exposure in the intervention group using a plancha woodstove but no effect on women’s lung function at 12–18 months in an intention-to-treat analysis.^28^ A subsequent exposure–response analysis did find a significantly decreased rate of decline with decreased exposure to CO.^29^ The use of a Patsari stove in rural Mexico was associated with a significantly decreased rate of lung function (FEV_1_) decline in women compared with those cooking on open fires (31 vs 62 mL), over 1 year of follow-up, but this effect was not observed on intention-to-treat analysis.^27^ This decrease in decline is comparable with that reported among ex-smokers, in the first year after quitting; their FEV_1_ trajectory showed half the rate of annual decline compared with those who continued to smoke ((mean±SD) 31±48 vs 62±55 mL).^30^ Our finding of FEV_1_ annual decline of 30.9 mL is consistent with the ranges seen in non-smokers from various studies.^11^

Our findings would suggest that low lung volumes seen in Malawian adults are not a result of accelerated decline in lung function, but more likely a failure to reach maximal lung volumes in early adulthood, either due to low lung function at birth or suboptimal lung growth during early childhood. Low birth weight and prematurity are of particular relevance in Malawi; the country has the highest rate of preterm birth worldwide, and intrauterine growth restriction, in both term and preterm infants, is common in low-income countries due to maternal factors including young maternal age, short-interpregnancy intervals and congenital infections.^31 32^ Adverse effects of prenatal exposure to household air pollution on infant lung function have been suggested by the recent GRAPHS trial in Ghana.^33^ The adverse effect of early respiratory infections on lung health in adulthood has long been recognised; such infections are common in ssA, particularly during the first year of life.^34 35^

Several studies have used CO levels as a proxy for particulate matter, which is challenging to measure in the field in low-resource settings. However, respirable particulate matter ≤2.5 µm (PM_2.5_) can reach the alveolar level in the lungs and is of greater interest when considering the adverse respiratory effects of air pollution. We found no association between PM_2.5_, CO or access to a cookstove and lung function. In keeping with findings from Peru, Nepal and Kenya, we observed poor correlation between CO and PM_2.5_ measurements and different explanatory covariates for the two pollutants in our exposure models.^36^ Although observed levels of exposure to both CO and PM_2.5_ exceeded WHO upper safety limits, the duration of these high exposures was brief and we speculate that adverse pulmonary effects are limited by the low intensity of exposure in rural Malawi where most cooking is done outdoors. Similarly, we found that current smoking was not associated with FEV_1_ in this population, likely reflecting the low intensity of tobacco use among smokers in this community; less than one-fifth of current or ex-smokers at baseline reported cigarette consumption of >10 pack years.

Strengths of our study include the collection of longitudinal lung function and personal air pollution exposure data in a rural cohort in one of the world’s poorest countries; high-quality spirometry performed by BOLD-certified technicians, with external quality control of traces by an independent expert reviewer. Limitations include potential recall bias and highly variable responses to questionnaires, and bias introduced by those not attempting spirometry or lost to follow-up. Participants performing spirometry were younger and hence, it is likely that spirometric abnormalities, such as obstruction, which are associated with increasing age are likely to be under-represented. Throughout the study the team struggled with cultural beliefs that older members of the community were “too weak” or “physically unable” to attempt spirometry. One-third of participants from baseline were lost to follow-up by the end of the study; we were unable to ascertain the reasons for this due to limitations of the data collected, but comparison of the demographic data for those who remained in the study at each phase suggested that men and those with better education were more likely to be lost, reflecting the more economically active, mobile sector of society. We recognise that 3 years is a relatively short time period to track longitudinal changes in lung function but believe we would more likely observe any effect of the intervention during the CAPS study period when the use of the cookstoves was actively supported by a repair and maintenance programme. Given that the rate in decline in lung function we observed over 3 years was consistent with the rate of decline seen in healthy adults in Europe and North America, it seems likely that this observation is accurate and that a longer period of follow-up would not have yielded additional useful rate of decline information.

In conclusion, in our cohort of adults living in rural Malawi, we observed (a) reduced FVC compared with Caucasian reference populations, similar in relative magnitude to what we previously reported in children living in the same communities, (b) no evidence of accelerated decline in FEV_1_ or FVC and c) no effect of access to cleaner-burning cookstoves on lung function decline. We suggest that future efforts to improve the lung health of those living in the poorest parts of the world should focus on antenatal and early childhood interventions to maximise lung growth and development. Further research is required to define the prognostic significance of reaching adulthood with suboptimal lung volumes, regardless of the comparative reference range in terms of morbidity, mortality and associated socioeconomic costs.
