# Supplementary material for: Non-communicable respiratory disease and air pollution exposure in Malawi: a prospective cohort study
Source: Thorax. 2020 Feb 20;75(3):220–6. doi: 10.1136/thoraxjnl-2019-213941 (PMC7063402; doi:10.1136/thoraxjnl-2019-213941)
Supplement: Supplementary data [file thoraxjnl-2019-213941supp001.pdf]

Online supplementary materials

Table S1. Availability of data (questionnaire, exposure monitoring and spirometry) for 1481 participants at baseline, first and second follow-up.

	Data available				Data not available		
	n	Mean age (SD)	Sex (% female)	Percent missing from baseline	n	Mean age (SD)	Sex (% female)
Questionnaire							
Baseline	1481	43.8 (17.8)	57.0	-	-	-	-
Follow-up 1	1090	43.6 (17.5)	60.6	26.4	391	44.5 (18.5)	46.8
Follow-up 2	989	44.0 (17.6)	62.9	33.2	492	43.5 (18.2)	45.1
Exposure monitoring							
Baseline	1029	44.0 (17.9)	57.1	30.5	452	43.4 (17.4)	56.6
Follow-up 1	830	44.0 (17.4)	61.8	44.0	651	43.7 (18.3)	50.8
Follow-up 2	811	44.4 (17.2)	62.3	45.2	670	43.2 (18.4)	50.6
Any measurement	1330	44.0 (17.7)	58.6	10.2	151	42.5 (18.1)	42.4
Multiple measurements	929	44.2 (17.5)	61.0	37.3	552	43.3 (18.1)	50.2
Spirometry							
Baseline	886	40.9 (15.3)	51.1	40.2	595	48.2 (20.2)	65.7
Follow-up 1	594	39.0 (14.1)	51.5	59.9	887	47.1 (19.2)	60.7
Follow-up 2	537	37.1 (13.8)	55.5	63.7	944	47.7 (18.6)	57.8
Any measurement	1086	40.2 (15.3)	53.3	26.7	413	53.2 (20.1)	66.6
Multiple measurements	654	38.4 (14.0)	52.0	55.8	827	48.1 (19.2)	60.9

Table S2. Likelihood ratio comparison of increasingly complex mixed-effects logCO response models

	Fixed effects parameters	*Comparison*	*LogLikelihood*	*Likelihood ratio test*	*df*	*p-value*
**1**	None	-	-4371.4			
**2**	Seasonality	1,2	-4344.8	53.2	2	<0.001†
**3**	Seasonality, sex	2,3	-4256.2	177.2	1	<0.001†
**4**	Seasonality, sex, age	3,4	-4256.1	0.1	1	0.768
**5**	Seasonality, sex, smoker	4,5	-4246.6	19.1	1	<0.001†
**6**	Seasonality, sex, smoker, cookstove	5,6	-4246.5	0.2	1	0.698

†Significant at 0.05 level and included in final model. Final fixed effects covariates highlighted in grey.

Table S3. Likelihood ratio comparison of increasingly complex mixed-effects log$PM_{2.5}$ response models

	Fixed effects parameters	*Comparison*	*LogLikelihood*	*Likelihood ratio test*	*df*	*p-value*
**1**	None	-	-6923.7			
**2**	Seasonality	1,2	-6923.2	1.1	2	0.574
**3**	Sex	1,3	-6915.4	16.7	1	<0.001†
**4**	Sex, age	3,4	-6914.7	1.3	1	0.256
**5**	Sex, smoker	3,5	-6913.6	3.4	1	0.064
**6**	Sex, cookstove	3,6	-6912.2	6.2	1	0.013†

†Significant at 0.05 level and included in final model. Final fixed effects covariates highlighted in grey.

Table S4. Comparison of increasingly complex mixed-effects FEV_1 response models

	Fixed effects parameters	*Comparison*	*LogLikelihood*	*Likelihood ratio test*	*df*	*p-value*
**0**	None	-	-13723	-	-	-
**1**	Time	0,1	-13704	38.7	1	<0.001†
**2**	Time, age	1,2	-13602	203.0	1	<0.001†
**3**	Time, age, sex	2,3	-13379	446.0	1	<0.001†
**4**	Time, age, sex, height	3,4	-13312	134.7	1	<0.001†
**5**	Time, age, sex, height, smoker	4,5	-13310	3.2	1	0.072
**6**	Time, age, sex, height, TB	4,6	-13299	25.5	1	<0.001†
**7**	Time, age, sex, height, TB, BMI	6,7	-13285	28.9	1	<0.001†
**8**	Time, age, sex, height, TB, BMI, years at school	7,8	-13284	1.3	1	0.248
**9**	Time, age, sex, height, TB, BMI, cookstove	7,9	-13285	0.14	1	0.706
**10**	Time, age, sex, height, TB, BMI, CO	7,10	-13285	0.08	1	0.777
**11**	Time, age, sex, height, TB, BMI, PM	7,11	-13284	2.1	1	0.146
**12**	Time, age, sex, height, TB, BMI, time*age	7,12	-13284	0.87	1	0.352
**13**	Time, age, sex, height, TB, BMI, time*sex	7,13	-13285	0.12	1	0.726
**14**	Time, age, sex, height, TB, BMI, time*TB	7,14	-13285	0	1	0.998
**15**	Time, age, sex, height, TB, BMI, time*PM	7,15	-13283	2.94	2	0.230
**16**	Time, age, sex, height, TB, BMI, time*CO	7,16	-13284	0.34	2	0.842

†Significant at 0.05 level and included in final model. Final fixed effects covariates highlighted in grey.
BMI = body mass index, CO = carbon monoxide, PM = fine particulate matter, TB = tuberculosis

Table S5. Comparison of increasingly complex mixed-effects FVC response models

	Fixed effects parameters	*Comparison*	*LogLikelihood*	*Likelihood ratio test*	*df*	*p-value*
**0**	None	-	-13912	-	-	-
**1**	Time	0,1	-13885	54.08	1	<0.001†
**2**	Time, age	1,2	-13862	45.96	1	<0.001†
**3**	Time, age, sex	2,3	-13560	604.00	1	<0.001†
**4**	Time, age, sex, height	3,4	-13454	212.11	1	<0.001†
**5**	Time, age, sex, height, smoker	4,5	-13454	0.08	1	0.775
**6**	Time, age, sex, height, TB	4,6	-13446	15.04	1	<0.001†
**7**	Time, age, sex, height, TB, BMI	6,7	-13435	22.69	1	<0.001†
**8**	Time, age, sex, height, TB, BMI, years at school	7,8	-13435	0.001	1	0.973
**9**	Time, age, sex, height, TB, BMI, cookstove	7,9	-13435	0.12	1	0.732
**10**	Time, age, sex, height, TB, BMI, CO	7,10	-13434	1.89	1	0.170
**11**	Time, age, sex, height, TB, BMI, PM	7,11	-13434	1.33	1	0.249
**12**	Time, age, sex, height, TB, BMI, time*age	7,12	-13433	3.34	1	0.068
**13**	Time, age, sex, height, TB, BMI, time*sex	7,13	-13435	0.08	1	0.78
**14**	Time, age, sex, height, TB, BMI, time*TB	7,14	-13434	0.79	1	0.37
**15**	Time, age, sex, height, TB, BMI, time*PM	7,15	-13434	2.70	2	0.259
**16**	Time, age, sex, height, TB, BMI, time*CO	7,16	-13434	1.96	2	0.376

†Significant at 0.05 level and included in final model. Final fixed effects covariates highlighted in grey.

BMI = body mass index, CO = carbon monoxide, PM = fine particulate matter, TB = tuberculosis

Text S1.

Regression equations for mixed-effects exposure models

CO model

$$logY_{ijt} = \alpha + b_{ijt}\beta_1 + c_{ijt}\beta_2 + d_{ijt}\beta_3 + e_{ijt}\beta_4 + U_i + V_{ij} + Z_{ijt}$$

where:

Y_{ijt} = exposure measurement for participant *i*, during 48-hr monitoring period *j*, on day *t*

$\beta_1, \beta_2, \beta_3, \beta_4$ = fixed effects parameter estimates

b = sex (female=1, male=0)

c = current smoker (yes=1, no=0)

$d = \cos\left(\frac{2\pi}{365} * \text{day of year}\right)$

$e = \sin\left(\frac{2\pi}{365} * \text{day of year}\right)$

$U_i \sim N(0, \sigma_u^2)$, random effect for the *i*th participant

$V_{ij} \sim N(0, \sigma_v^2)$, random effect for *j*th 48-hour monitoring period in the *i*th participant

$Z_{ijt} \sim N(0, {\sigma_x}^2)$, error term associated with the *t*th measurement in the *j*th 48-monitoring period for the *i*th participant.

$PM_{2.5}$ model

$$logY_{ijt} = \alpha + b_{ijt}\beta_1 + c_{ijt}\beta_2 + U_i + V_{ij} + Z_{ijt}$$

where:

Y_{ijt} = exposure measurement for participant *i*, during 48-hr monitoring period *j*, on day *t*

β_1, β_2 = fixed effects parameter estimates

b = sex (female=1, male=0)

c = access to cookstove (yes=1, no=0)

$U_i \sim N(0, \sigma_u^2)$, random effect for the *i*th participant

$V_{ij} \sim N(0, \sigma_v^2)$, random effect for the *j*th 48-hour monitoring period in the *i*th participant

$Z_{ijt} \sim N(0, {\sigma_x}^2)$, error term associated with the *t*th measurement in the *j*th 48-monitoring period for the *i*th participant.

Regression equations for mixed-effects lung function (FEV_1 and FVC) models

$$Y_{it} = \alpha + b_{it}\beta_1 + c_{it}\beta_2 + d_{it}\beta_3 + e_{it}\beta_4 + f_{it}\beta_5 + g_{it}\beta_6 + U_i + Z_{it}$$

where:

Y_{ijt} = lung function measurement for participant *i*, on day *t*

$\beta_1, \beta_2, \beta_3, \beta_4, \beta_5, \beta_6$ = fixed effects parameter estimates

b = time in years

c = age in years

d = sex (female=1, male=0)

e = height in cm

f = previous tuberculosis (yes=1, no=0)

g = Body Mass Index in kg/m^2

$U_i \sim N(0, \sigma_u^2)$, random effect for the *i*th participant

$Z_{it} \sim N(0, {\sigma_x}^2)$, error term associated with the *t*th measurement for the *i*th participant.
